# Investigating the Role of Islet Cytoarchitecture in Its Oscillation Using a New β-Cell Cluster Model

**DOI:** 10.1371/journal.pone.0000983

**Published:** 2007-10-03

**Authors:** Aparna Nittala, Soumitra Ghosh, Xujing Wang

**Affiliations:** Max McGee National Research Center for Juvenile Diabetes, Human and Molecular Genetics Center, Medical College of Wisconsin, Children's Research Institute of the Children's Hospital of Wisconsin, Milwaukee, Wisconsin, United States of America; University of Auckland, New Zealand

## Abstract

The oscillatory insulin release is fundamental to normal glycemic control. The basis of the oscillation is the intercellular coupling and bursting synchronization of β cells in each islet. The functional role of islet β cell mass organization with respect to its oscillatory bursting is not well understood. This is of special interest in view of the recent finding of islet cytoarchitectural differences between human and animal models. In this study we developed a new hexagonal closest packing (HCP) cell cluster model. The model captures more accurately the real islet cell organization than the simple cubic packing (SCP) cluster that is conventionally used. Using our new model we investigated the functional characteristics of β-cell clusters, including the fraction of cells able to burst *f*_b_, the synchronization index λ of the bursting β cells, the bursting period *T*_b_, the plateau fraction *p*_f_, and the amplitude of intracellular calcium oscillation [*Ca*]. We determined their dependence on cluster architectural parameters including number of cells *n*_β_, number of inter-β cell couplings of each β cell *n*_c_, and the coupling strength *g*_c_. We found that at low values of *n*_β_, *n*_c_ and *g*_c_, the oscillation regularity improves with their increasing values. This functional gain plateaus around their physiological values in real islets, at *n*_β_∼100, *n*_c_∼6 and *g*_c_∼200 pS. In addition, normal β-cell clusters are robust against significant perturbation to their architecture, including the presence of non-β cells or dead β cells. In clusters with *n*_β_>∼100, coordinated β-cell bursting can be maintained at up to 70% of β-cell loss, which is consistent with laboratory and clinical findings of islets. Our results suggest that the bursting characteristics of a β-cell cluster depend quantitatively on its architecture in a non-linear fashion. These findings are important to understand the islet bursting phenomenon and the regulation of insulin secretion, under both physiological and pathological conditions.

## Introduction

Insulin, secreted by pancreatic islet β cells, is the principal regulating hormone of glucose metabolism. Defects in insulin release or its utilization lead to scores of metabolic disorders, including diabetes. In normal human individuals, insulin is secreted in a pulsatile fashion, and the plasma insulin exhibits an oscillatory pattern, both at basal and under stimulation [1], [2]. Oscillatory insulin is critical to normal glycemic control; loss of oscillation desensitizes the insulin receptors of target tissues and contributes to insulin resistance [1], [3], [4]. This is a general feature of endocrine glands to signal their remote target organs by pulsatile rather than continuous hormone secretion in order to avoid down-regulation of target tissue responses [5]. In patients with deficient endogenous insulin secretion, pulsatile insulin delivery was proven to be superior than continuous or bolus delivery [6]–[8]. The importance of insulin pulsatility in glucose regulation is further indicated by the loss of it observed in patients of both type 1 diabetes (T1D) and type 2 diabetes (T2D) [1], [9], which together constitute more than 95% of all diabetes cases. More interestingly, the alterations in insulin oscillation have also been observed in individuals at risk (first degree relatives) for both T1D [10] and T2D [11], [12], indicating its potential in developing early disease markers. A quantitative understanding of the regulation of insulin pulsatility is important to the understanding of glucose metabolic control, and to diabetes research.

Insulin-secreting β cells, which comprise about 1% of the total pancreatic cell populations, are not uniformly distributed across the pancreas; rather they are organized in the islet of Langerhans. Each human islet has ∼10^3^ cells, with the majority of them (∼50–70%) being β cells. This hierarchical organization of β-cell mass is fundamental to their pulsatile insulin release, which is driven by the electrical burst of the cell membrane. Although theoretically single isolated β cells can burst, and can be induced *in vitro* to release insulin under tightly controlled conditions, they respond poorly to glucose and the bursting usually comprises erratic spiking that is highly sensitive to noise [13]–[19]. In contrast, the insulin release profile from β cells in intact islets is much more regular and robust against noise [20]–[22]. Experimental data have demonstrated that β cells within the same islet exhibit synchronous oscillation [22]–[24], leading to an appropriate glucose dose response. Extensive mathematical modeling and computational simulation of the islet oscillation has been carried out in the past several decades [2], [13]–[15], [25]–[32]. Among the models that have been proposed, the heterogeneity hypothesis is gaining acceptance [15], [27], [32]. The pancreatic islet β cells are highly heterogeneous in their biophysical properties including size, membrane capacitance, channel density, and channel conductance, etc. This results in diverse responses under the same condition, often with the majority of the β cells outside the narrow bursting regime. According to this model, however, in a coupled cluster, the over- and under-active β cells will cooperate through intercellular gap junctional coupling, and together they are able to reach more pronounced, synchronized bursts. An alternative hypothesis, proposed by the same group, is the channel sharing theory [13], [26], [33]. It postulates that the ionic current fluctuations arising from channel-gating stochasticity can often disrupt the bursting regularity from individual β cells. In a cluster such fluctuations are largely neutralized through gap junctional coupling among β cells, leading to coordinated bursting of the whole cluster. Later it was found that even within the channel sharing hypothesis, heterogeneity is needed to produce regular bursting [34]. In this study we will adopt the heterogeneity hypothesis.

Despite much works, a quantitative investigation of the islet oscillation with respect to the cytoarchitectural organization of its β-cell mass has not been performed. The islet is a highly organized functional micro-organ with defined size and structure, intercellular coupling, and autocrine and paracrine regulations. As the pancreatic islet is not readily accessible in humans, in vivo study of β-cell function primarily relies on animal models. However, recent studies revealed architectural differences between human and animal islets [35], [36], and it is still not clear whether and how these physical differences translate functionally.

In this study we investigate, for the first time, the role of islet cytoarchitecture in the coordination of β-cell bursting. The architectural parameters include the number of β cells in a cluster *n*_β_, number of inter-β cell couplings of each β cell *n*_c_, and the intercellular coupling strength *g*_c_. Within a β-cell cluster, the fraction of β cells (*f*_b_) that are able to establish regular bursting patterns, and the degree of bursting synchronization (λ) among them, are clearly important to the insulin release function of the cluster. The plateau fraction (*p*_f_), which is defined to be the relative time spent in the active phase divided by the total burst interval [37], is another important parameter. The amount of insulin released is thought to be proportional to the plateau fraction [37]. Other parameters that have been speculated to be indicative of function include the bursting period (*T*_b_) and the amplitude of the intracellular calcium oscillation ([*Ca*]) [14]. In this study we will quantitatively evaluate these five functional measures and their changes with varying values of *n*_β_, *n*_c_, and *g*_c_. To facilitate such investigation, we introduce a new Hexagon Closest Packing (HCP) cell cluster model for β cells in an islet. The HCP architecture is significantly closer to a real islet than the Simple Cubic Packing (SCP) scheme that has been conventionally utilized till now.

## Results

### Development of the HCP β-cell cluster model

#### The HCP β-cell cluster

**Figure 1:**
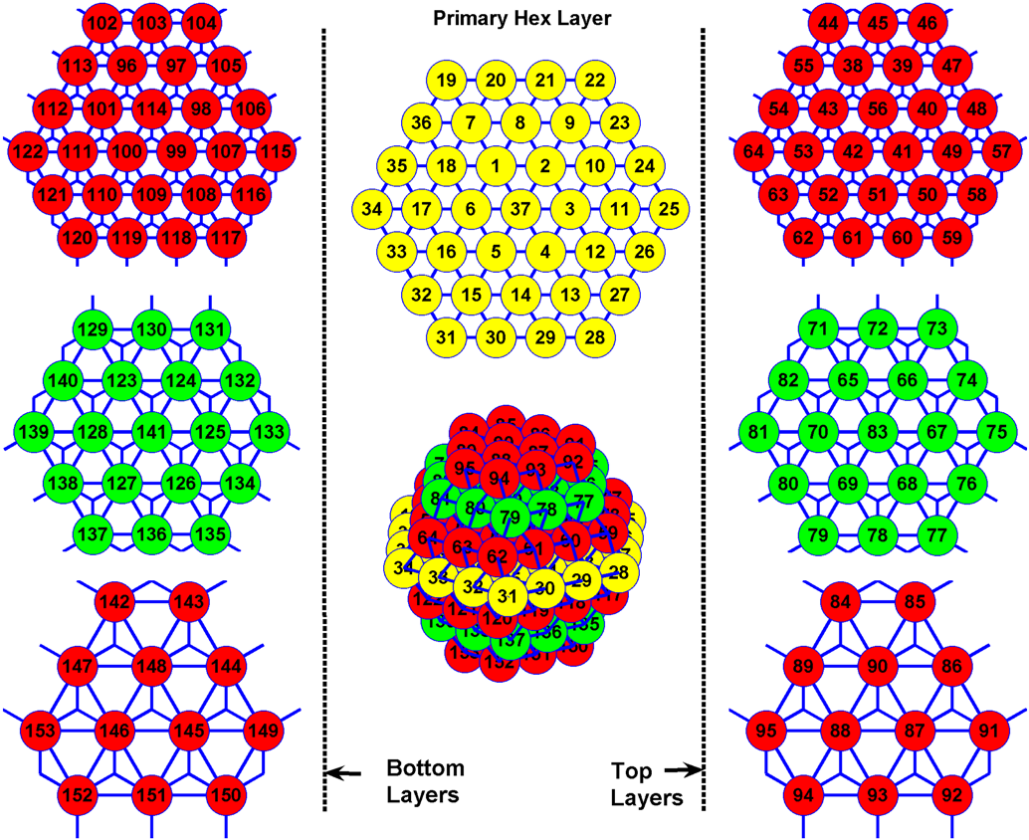
Cell labeling in a HCP cluster of *n* = 4.

The structural details of the HCP and SCP lattices are given in the method section. To organize β cells in a cluster, existing models till now utilized the SCP lattice, which gives *n*_c_ = 4 in 2D and *n*_c_ = 6 in 3D. While examining the architecture of real islets, we realized that this protocol significantly under-estimates *n*_c_. In addition, this does not give us much room to investigate the functional consequence of islet architecture with varying *n*_c_ values, or the consequence of non-β cells or β-cell death. In this study we introduced the HCP lattice to simulate β-cell clusters. This is based on the idea that a biological cell can be approximated by a sphere, and spheres can be packed more efficiently using HCP (the most compact packing for spheres) than using SCP. In vitro experiments that studied clusters of β cells also indicated that the hexagonal lattice is a much better approximation to the cluster structure [38], [39]. With a HCP cluster the number of intercellular couplings of each β cell is *n*_c_ = 6 in 2D and *n*_c_ = 12 in 3D. The arrangement of β cells in a HCP cluster and the setting up of their coupling terms 
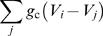
 is not as straightforward as in the case of a SCP cluster. We have developed an algorithm, described in the method section, to carry out this task in an automatic fashion. Figure 1 shows a HCP cluster with edge size *n* = 4, and the cell numbering throughout its layers. Solid lines represent the intercellular couplings.

#### Synchronization of bursting cells

**Figure 2:**
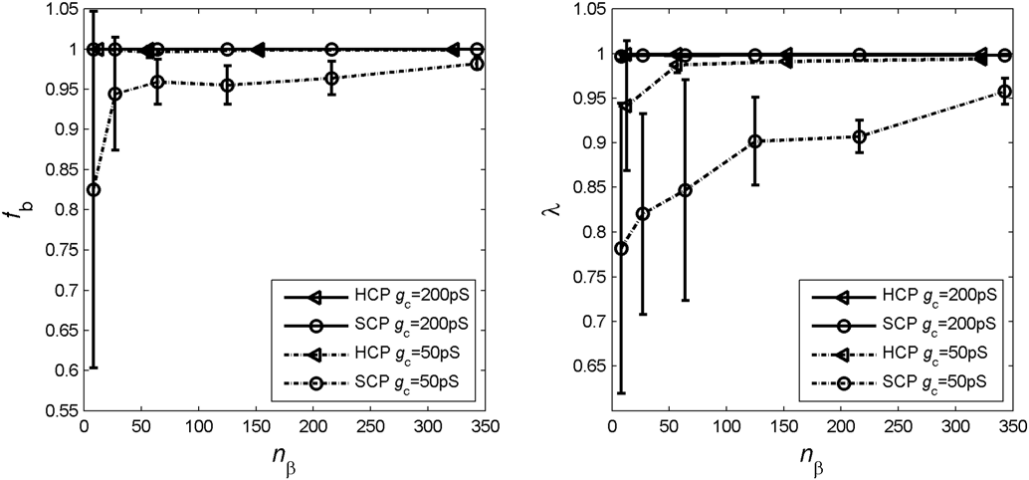
The fraction of β cells able to burst *f*_b_, and their synchronization λ, are less influenced by cluster size *n*_β_ in HCP clusters. Plotted are mean and standard deviation of the corresponding 10 replicate clusters at each condition.

We first compared the fraction of burster cells *f*_b_ and their synchronization index λ for all SCP (SCP-8, -27, -64, -125, -216, and -343) and HCP (HCP-13, -57, -153 and -323) clusters we have simulated, at different gap junctional coupling strength from *g*_c_ = 25 pS to *g*_c_ = 1000 pS. We find that at or above normal physiological conditions of *g*_c_∼150–250 pS [14], [40], [41], the SCP and HCP clusters did not differ much and all cluster sizes exhibited a high fraction (>99%) of well synchronized burster cells (λ>0.95). Figure 2 presents the results at *g*_c_ = 200 pS. It is known that under pathological conditions, the intercellular coupling can be significantly impaired. For example, chronic hyperglycemia can reduce the gap junctional conductance between cells [42]–[45]. A particular case, when *g*_c_ reduces to 50 pS, is also plotted in figure 2 for both cluster types. Over 99% of the β cells in HCP clusters are still able to burst, while this number dropped significantly in the SCP clusters with the smaller ones affected the most. Additionally, the synchronization among the burster cells is also significantly less in the SCP clusters, especially the smaller SCP clusters.

**Figure 3:**
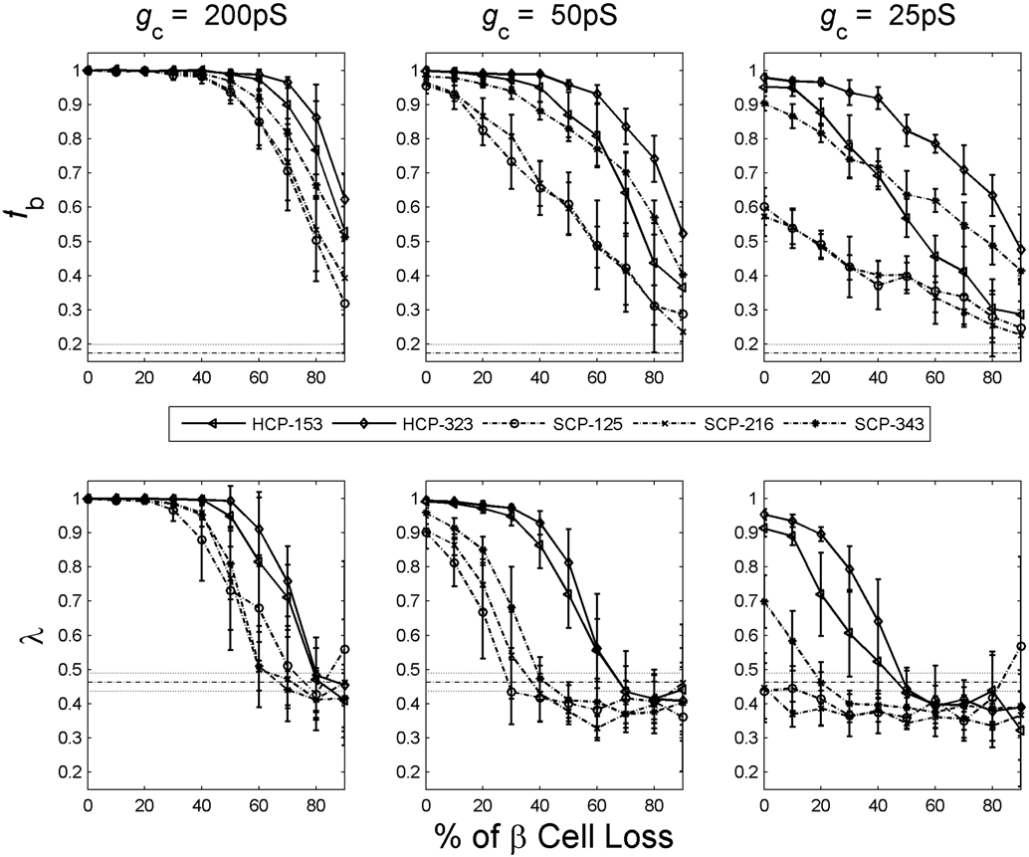
The HCP clusters are more robust against perturbations to its architecture than the SCP clusters. At normal coupling strength of *g*_c_ = 200 pS (left columns), both cluster types are robust against up to 50% β-cell loss in the cluster. When the coupling is compromised with reduced *g*_c_ values (middle and right columns), the SCP clusters are more affected by β-cell loss. Dashed lines are the mean and the 2 standard deviations (SD) of completely uncoupled β cells.

Under pathological conditions including diabetes, loss of β-cell mass usually occur [46]. It can be a mere loss at peripheral that affects only the value of islet size *n*_β_. More often, the β-cell loss occurs across the islet, which affects the values of both *n*_β_ and *n*_c_. We have compared the two cluster types with varying percentages of β cells randomly removed from the cluster. In figure 3, we present results at normal and impaired gap junctional coupling *g*_c_. At normal coupling strength of *g*_c_ = 200 pS (left column), both SCP and HCP are fairly robust against loss of β cells. With up to 50% of β cells lost, over 90% of the remaining cells in the clusters are still able to burst with significant synchronization of λ>0.7.

A close look of the figure reveals that the HCP clusters are more robust than SCP ones, especially when β-cell loss is over 50%. This difference is more pronounced and can be observed sooner when the gap junctional coupling is impaired. Specifically at *g*_c_ = 50 pS (figure 3, middle column), a HCP cluster can tolerate more than 50% of β-cell loss, while 30% β-cell loss will completely disrupt the bursting synchronization of an SCP cluster; at *g*_c_ = 25 pS (figure 3, right column), even with 50% β-cell loss, the majority of remaining cells in the HCP clusters are still able to burst synchronously, whilst the SCP clusters would not endure any β-cell loss (with the exception of SCP-343, at ≤10% β-cell loss).

#### Other functional parameters

**Figure 4:**
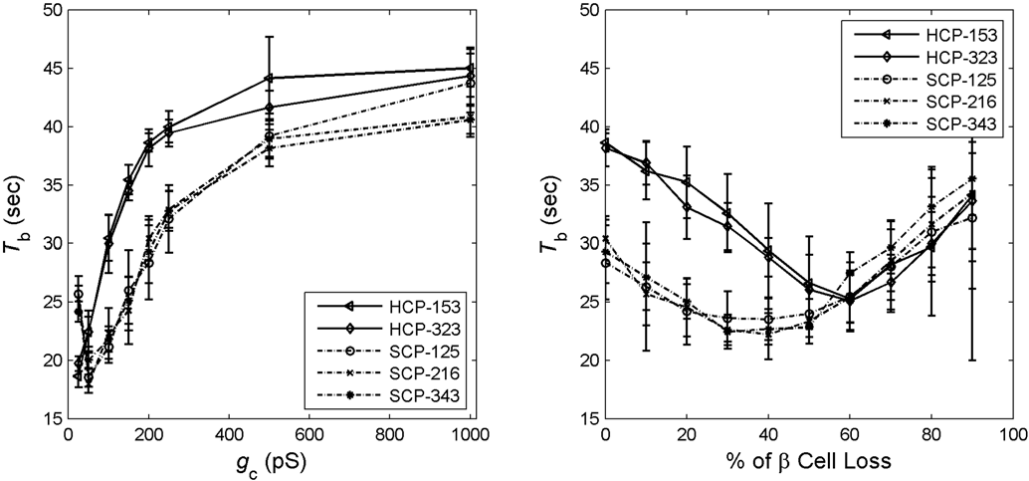
Comparison of the bursting period *T*_b_ between HCP and SCP β-cell clusters.

We have also compared the HCP and SCP clusters in terms of the other three functional measures: bursting period *T*_b_, plateau fraction *p*_f_, and calcium amplitude [*Ca*]. We found that under normal conditions, HCP β-cell clusters burst with significantly longer period (figure 4). This is true even when the cell cluster is under significant perturbation, with impaired intercellular coupling as low as *g*_c_ = 50 pS and β-cell damage up to 50%. When we examined *p*_f_ and [*Ca*], we did not see significant difference between the two types of clusters, either at normal or under compromised cluster architectural parameter values (data not shown).

### Function of a β-cell cluster versus its architectural parameters

In this session we examined in detail the quantitative dependence of the 5 functional measures (*f*_b_, λ, *T*_b_, *p*_f_, and [*Ca*]) on the three architectural parameters (*n*_β_, *n*_c_ and *g*_c_), using our HCP model of β-cell cluster. In real human islets, there are significant amounts of non-β cells (up to 30–50%), which do not couple or synchronize with β cells. Studies that profiled Ca^2+^ concentration within intact islets found that the oscillation in non-β cells were completely asynchronous, both with respect to each other and to β cells [23]. We therefore carried out our investigation with 30–50% of the cells randomly removed from coupling with the rest of the cells in the cluster.

#### Size of a β-cell cluster *n*_β_

**Figure 5:**
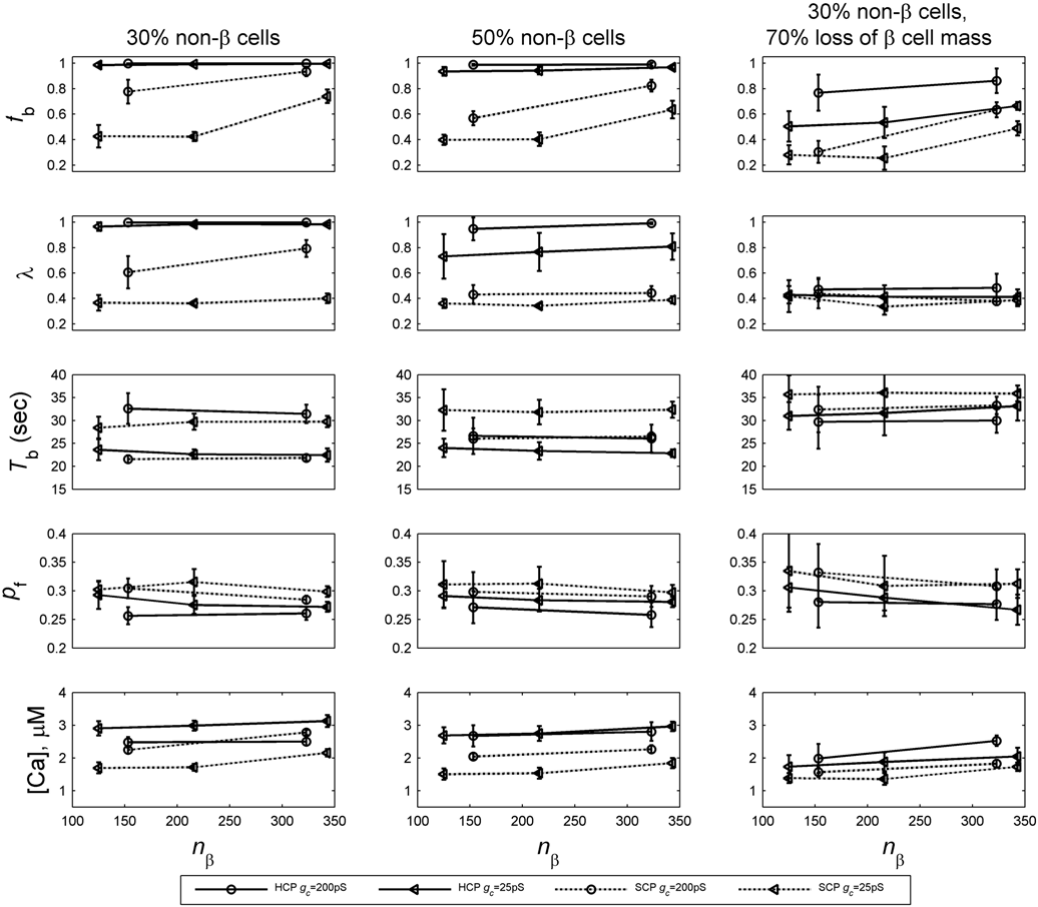
Function of a β-cell cluster versus its size *n*_β_. Left and middle columns: at normal coupling strength and no loss of β cells, no significant dependence is observed in the HCP β-cell clusters (solid lines). This is true even when the intercellular coupling is impaired to a very low value of 25 pS. Right column: when there is extensive β-cell loss at 70%, marginal functional gain is observed in *f*_b_ and [*Ca*].

Under normal coupling strength ∼150–250 pS [41], no significant functional dependence on cluster size *n*_β_ has been observed for β-cell clusters with more than ∼100 cells. Figure 5 (left and middle columns) depicts simulation results at *g*_c_ = 200 pS. Evidently none of the 5 functional measures showed notable dependence on cluster size *n*_β_. We then examined if larger clusters have a functional advantage when the intercellular coupling or functional β-cell mass is compromised. We found that advantage of larger clusters is only notable under extreme pathological conditions. In the same figure, we also included results at *g*_c_ = 50 pS, and when there is 70% loss of β cells (right column). Moderate functional gain with increasing *n*_β_, primarily in terms of improved *f*_b_ and λ, is observed. Results of the SCP clusters, also included in figure 5, indicate the same conclusion.

The benefit of increasing *n*_β_ is most notable for very small clusters with *n*_β_<100. In the previous section where we compared the SCP and HCP clusters, smaller clusters with less than 100 β cells (SCP-8, SCP-64, HCP-13 and HCP-57) were also simulated (figure 2). These results also indicated little dependence on cluster size. Only when coupling is impaired, there is moderate functional gain with increasing cluster size (dashed lines in figure 2).

#### Number of intercellular couplings per β cell *n*_c_

As we have pointed out earlier non-β cells in real human islets likely do not couple or synchronize with β cells [23]. Their presence will make the effective *n*_c_ value less than the number of the nearest neighbors each β cells has (as not all of them are β cells). 30% non-β cells translates to an effective *n*_c_ = 8.4 in our HCP model, and 50% non-β cells means *n*_c_ = 6. Pathological loss of β-cell mass can also lead to reduced *n*_c_. Therefore it is of interest to determine the functional role of *n*_c_.

**Figure 6:**
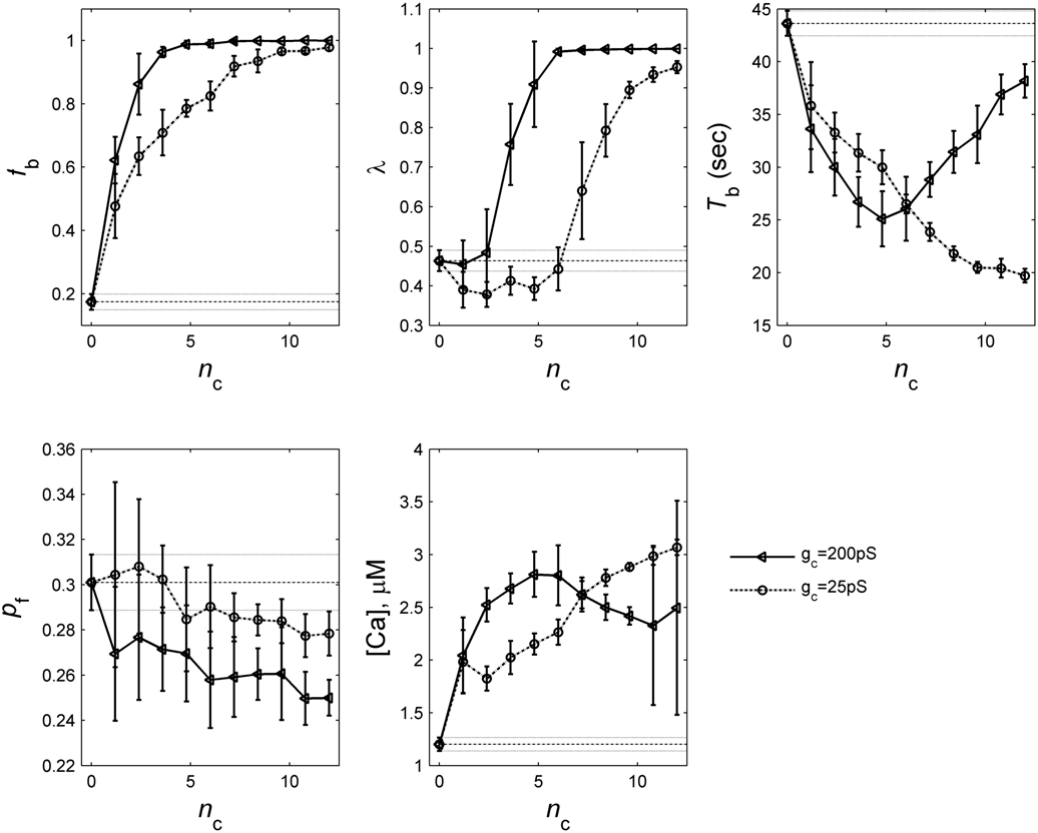
Function of β-cell clusters versus *n*_c_. Plotted are results of a HCP-323 cluster. Dashed lines are the basal level values and 2 SD that would be expected from a cluster of un-coupled β-cells

Intuitively one would expect that more intercellular coupling is desirable for the heterogeneous β-cell population in order to coordinate and synchronize their bursting and insulin release. We have found that this is indeed the case. Figure 6 gives the results of the 5 bursting measures for a HCP-323 β-cell cluster at normal and impaired coupling strength of *g*_c_ = 200 pS and *g*_c_ = 25 pS, respectively. We have also included in the figure, results for a cluster of totally uncoupled β cells (*n*_c_ = 0 or *g*_c_ = 0, dashed lines). The results of the two synchronization measures, *f*_b_ and λ, are particularly revealing. Under normal conditions each β-cell on average needs to be coupled to at least 3 other β cells (*n*_c_>2.4), in order to reach synchronized bursting of the cluster. The bursting coordination improves with increasing value of *n*_c_. However, this improvement plateaus at around *n*_c_ = 6, a value that is about half of its up-limit in a HCP cluster. The simulation results for other clusters with more than 100 β cells (HCP-153 and SCP125, -216, -343) are quantitatively similar (data not shown). Interestingly the amount of β-cell couplings in a normal HCP β-cell cluster with 30–50% of non-β cells (as human islets do) is *n*_c_∼6–8.4. This gives the β-cell cluster some room to tolerate perturbation to its architecture, namely, a small amount of random β-cell loss will not lead to significant alteration of the cluster function. When the gap junctional coupling is impaired, it takes much higher values of *n*_c_ to reach a good bursting synchronization among β cells. At *g*_c_ = 25 pS, a ∼10 fold reduction from the normal strength, synchronized bursting can only be achieved with *n*_c_>6. These results demonstrate that a β-cell cluster with a higher-*n*_c_ architecture will be more robust against pathological perturbations.

#### Intercellular coupling strength *g*_c_

**Figure 7:**
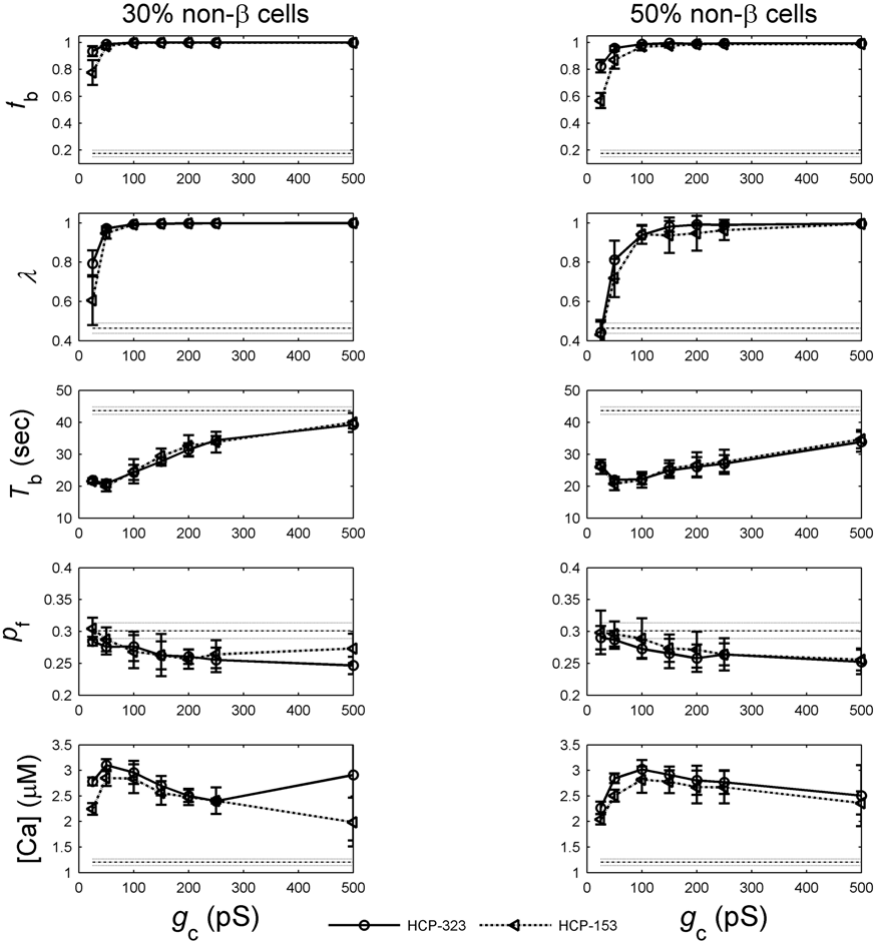
Function of β-cell clusters versus intercellular coupling strength *g*_c_.

The intercellular coupling strength is known to be a critical parameter for normal islet function [14], [43], [47]. Impaired gap junctional coupling between β cells will compromise their glucose sensitivity and insulin release functionality [48], [49]. We have simulated β-cell clusters at varying coupling strength from 25 pS to 1000 pS. We find that at low values below the normal physiological range of increased *g*_c_ generally leads to higher *f*_b_ and λ. Figure 7 presents the results at both 30% and 50% of non-β cells. Evidently, the functional gain in *f*_b_ and λ plateaus around ∼200 pS.

The other three functional measures including *T*_b_, *p*_f_ and [*Ca*] also showed dependence on *g*_c_, though the interpretation is less obvious. The relationship is not always monotonic (figure 7). Specifically, the calcium amplitude profile exhibited an initial rise with increasing value of *g*_c_, it then peaked around a value within the normal physiological range of *g*_c_ and then drops afterwards. This phenomenon has also been observed by others [14]. In general there has not been enough study or a priori knowledge of these three parameters to presume what values are functionally desirable. Of interest is the fact that when *g*_c_ is beyond the normal physiological value range (*g*_c_>∼250 pS), dependence on *g*_c_ is much less marked for all parameters and even diminishing for some.

#### Loss of β-cell mass

**Figure 8:**
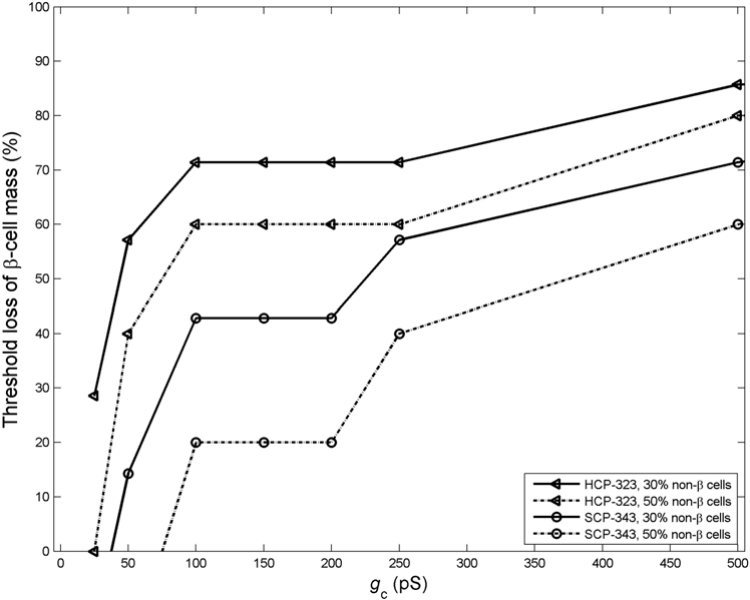
Threshold of β-cell loss that would lead to complete loss of bursting synchronization.

During the disease progression for all major forms of diabetes loss of β-cell mass has been observed [46]. The alteration sometimes could be merely a reduction in islet size *n*_β_, but more often more profound changes to the islet cytoarchitecture occur. For example, in type 1 diabetes the infiltrating immune cells spread from peripheral islet vessels to the centre of a given islet, causing β-cell apoptosis across the islet [50], [51]. In this situation both *n*_β_ and *n*_c_ are reduced, more significantly so for *n*_c_. For this reason we have examined the impact to the cluster bursting pattern when there is random β-cell loss across the cluster. Both *f*_b_ and λ fall with increasing loss of β cells (data not shown). The results are similar to that presented in figure 3, where we compared the SCP and HCP cluster at 0% of non-β cells. We are specifically interested in the threshold loss of β-cell mass that would lead to functional failure. As the coordination and synchronization of the β-cell bursting within an islet is fundamental to regulated, glucose dose-dependent insulin release, we investigated this problem from the perspective of β-cell bursting synchronization. We first simulated and determined the degree of synchronization that would be expected by chance for a cluster of uncoupled β cells. We then assumed that a β-cell cluster would not be able to function normally if its λ falls below 2 SD of this basal level. Using this criterion, we investigated the threshold loss of β-cell mass that would result in functional failure, under varying values of *g*_c_. Around physiological values of *g*_c_ (150–250 pS), at 30% non-β cells, we found that HCP β-cell clusters can function with majority, up to 70%, of its β-cell mass lost. The result of *g*_c_ = 200 pS is given in figure 8. This is consistent with the laboratory and clinical findings. Laboratory studies have shown that islets are tolerant of substantial β-cell loss, and can function normally with majority of β cells either dead or non-functioning [48]. Clinically it is believed that in type 1 diabetes disease onset occurs with up to 70–90% of β cells are destroyed [52]. In contrast, the SCP clusters do not have such robustness, the threshold loss is only 40% at *g*_c_ = 200 pS (figure 8). If the intercellular coupling is impaired with a low *g*_c_ value, the function of the β-cell cluster is much less robust against β-cell damages. At *g*_c_∼25 pS, a 30% of β-cell loss would completely disrupt the bursting coordination of a HCP β-cell cluster.

## Discussion

In this first effort to quantitatively investigate the role of islet cytoarchitecture with respect to its pulsatile bursting, we examined three structural parameters of β-cell clusters: cluster size *n*_β_, the number of intercellular couplings each β cell has *n*_c_, and the intercellular coupling strength *g*_c_. To enable a more realistic mathematical investigation of islet architecture, we introduced a new β-cell packing model, based on the HCP lattice structure, to replace the SCP model that is conventionally used. The HCP cluster is a much closer approximation of the β-cell arrangement in real islets [38], [39], and more accurately captures the degree of intercellular coupling *n*_c_. We found that the HCP β-cell clusters are functionally more desirable than the SCP clusters, as evidenced from the higher fractions of burster cells (*f*_b_), better synchronization (λ), and longer bursting periods (*T*_b_). In addition, the HCP clusters are significantly more robust against architectural perturbations, including presence of non-β cells, β-cell damage and impaired gap junctional couplings. Further, the proposed HCP architecture is able to explain why islets can function with majority of β cells lost. In contrast, the SCP model would require majority of its β cells to be intact for the β-cell cluster to burst regularly. Therefore we believe that the HCP model is a better one to study the islet bursting phenomenon. In this study, we utilized our HCP model to investigate quantitatively the dependence of islet bursting on its architecture. We have found that the functional measures, including *f*_b_, λ, *T*_b_, *p*_f_, and [*Ca*], depend exquisitely on the three architectural parameters *n*_β_, *n*_c_ and *g*_c_. As the ability to reach synchronized bursting is fundamental to the regulation of islet insulin release, and its role in islet function is much better understood than the other three parameters (*T*_b_, *p*_f_, [*Ca*]), our investigation focused more on the evaluation of *f*_b_ and λ.

### Is there an optimal islet size?

When judged by the fraction of burster β cells *f*_b_ and the degree of their synchronization λ, our simulation results suggest that the very small β-cell clusters (*n*_β_<50) are not functionally stable (figure 2, large standard deviations). They are also very sensitive to structural perturbations including β-cell damage and intercellular coupling impairment. When the size increases, initially there is functional gain with increasing cluster size (figure 2). This is manifested in improved *f*_b_ and λ values, and robustness against β-cell death. However the functional gain plateaus when the β-cell number reaches above ∼100–200 cells (figure 5), suggesting that very large β-cell clusters are not necessarily more desirable.

In reality the islet size is also limited by other factors. In our present modeling and simulation, we did not consider the non-uniform distribution of external stimuli such as the intra-islet glucose concentration. In rodent islets, it is believed the blood vessel goes to the center of the islet and blood flows from the center out. The architecture and microcirculation of human islets are believed to be more complex [35], [36], and may contain several sub-compartments of β-cell clusters organized around the micro vascularization within the islet. This would lead to more complicated pattern of non-uniformity in intra-islet glucose concentration, serving as a confounding factor on top of the heterogeneous biophysical properties of β cells. In vitro studies have demonstrated that such a glucose gradient can result in an attenuating propagation wave of intra-islet calcium, resulting in the inactivity of β cells that are far from the center. Several other problems that may be a concern in large islets include the oxygen supply, the microvascular circulation that are needed to support cell metabolism, and the paracrine relationship between cells, etc.

In vitro functional studies of both human and animal islets have revealed that larger islets are less efficient at insulin release than smaller islets under basal and stimulatory glucose levels [53], [54]. In the work by Lehmann et al [54], human islets were separated into two groups according to their diameter: small islets, 50–150 um; and large islets, 150–300 um. When normalized with islet volume (termed IEQ, islet equivalent), basal and stimulated insulin secretion was twice as high in small islets compared with large islets. Similar findings were observed in [53] for rat islets. In these studies, the large islets contain ∼10^3^ cells and the small islets ∼10^2^ cells. These studies also examined the transplantation outcome and clear superiority of the small islet group was observed [53], [54]. These indicate that there could be a functional compromise when the islet size gets too big *in vivo*.

Mammalian species span several orders of magnitude in terms of body weight, with commensurate demand on insulin and hence total endocrine pancreatic mass. Interestingly, the increased endocrine mass in larger mammals is manifested in the form of increased number of islets, rather than significantly larger islets. For example, the body weight of an average human being is around 1,000 fold of rodents, and the total endocrine β-cell mass is roughly in the same proportion. On average, humans have ∼10^6^ islets, whilst rodents ∼10^3^, a 1,000 fold difference. In contrast their islet sizes are within the same order of magnitude at ∼10^2^–10^3^. This phenomenon also indicates a possible functional optimal range for islet size at *n*_β_∼10^2^–10^3^, and different species may have all arrived at values within this range through evolution.

It is known that under certain physiological or pathological conditions, such as during pregnancy and obesity, expansion of islet size (*n*_β_) has been observed [46]. It is still not clear if the expansion, assumed to have occurred in order to compensate for the increased demand, is really able to adequately address the need, and if it has played a role in the increased risk for diabetes that these individuals experience. These together with our modeling findings suggest that the quantitative relationship between islet function and its size deserves more attention.

### The importance of team work

In a real islet the effective number of inter-β cell coupling *n*_c_ that each β cell has is less than the number of its nearest neighbors. Further the value *n*_c_ is readily influenced by physiological or pathological changes such as presence of non-β cells, or β-cell damage. In this study, the role of *n*_c_ in islet function was, *for the first time*, quantitatively investigated. We found that at normal coupling strength (*g*_c_∼200 pS), each β cells on average needs to be coupled to at least 3 (*n*_c_>2.4, figure 6) other β cells in order to coordinate their bursting throughout the whole cluster. If the intercellular coupling strength is weak, then this number needs to be even higher.

The investigation of *n*_c_ as an islet architectural parameter is also important to translational research. Much of our past understanding of islet function and structure was obtained from rodent models, where most β cells are clustered around a center core, surrounded by a layer of α and δ cells. Recent studies have revealed a cytoarchitectural difference between human and rodent islets [35], [36]. It was found that human islets on average contained proportionally fewer β cells, ∼55% versus 70–80% in rodents [35], [36]. In addition, throughout human islets β cells are more intermingled with the other islet cell types (mainly α and δ cells). It was estimated that about 70% of the β cells exclusively associate with β cells in rodent islets (corresponding to a *n*_c_∼8.4 HCP cluster), whilst in human islets, this number can be as low as 30% [35], [36] (*n*_c_∼3.6). These reports suggest that rodent islets may have much higher *n*_c_ than human ones. It might still be arguable if the apparent cytoarchitectural difference is fundamental to function. Concerns that have been raised include the fact that human islets were usually obtained under different pathological conditions, and that human islets may be of a composite compartmental structure with each compartment organized in the same architectural principle of a single rodent islet (private communication with experts in the field). Measurements with human islets did show subregions of β cells synchronization, but not across the whole islet [55]. More studies are obviously needed to settle these issues. However, we would like to point out that a quantitative investigation of islet function versus its structural parameters will provide insights to the cytoarchitectural organization principle of β-cell mass, and whether there are fundamental differences across different species. Understanding these issues will be highly valuable to translational studies using animal islets.

### The strength of bond

The strength of the intercellular coupling is another important islet architectural parameter. Inhibition of gap junctions led to loss of glucose sensitivity in intact rodent islets [48]. In this study our simulation results suggest that there is an optimal value range for function around 100–200 pS, beyond which there is little functional gain with increasing values of *g*_c_. Similar results have been previously observed by others in their simulation studies of islet oscillation [14]. This range is around the values observed in normal rodent islets (213+/−113 pS) [41]. Very few human islet measurements are available at this time.

The intercellular couplings are mediated through channels located at gap junctions. Gap junctions are specific membrane structures consisting of aggregates of intercellular channels that enable the direct exchange of ions and small metabolites (<1 kD) [56]. Such intercellular channels result from the association of two hemichannels, named connexons, which are separately contributed by two adjacent cells. Each connexon is an assembly of six transmembrane connexins, encoded by a family of genes with more than 20 members. The coupling strength depends on the number of gap junctions, as well as the number and the physiological state of their constituting subunit connexons. Extra energy (to make connexons) is required to increase the value of *g*_c_. It is therefore not surprising that normal islets function at a *g*_c_ value beyond which little functional gain can be achieved. Further it has been found that glycemic condition directly affects the transcription level of connexins [42] and hence the value of *g*_c_. Diabetes therapeutic drugs that were designed to target at intercellular cAMP levels or K_ATP_ channels, are often found to also modulate the value of *g*_c_ [57]. These all indicate the importance of the β-cell gap junctional coupling in normal islet function, and more work is needed in this area.

### Pathological alterations to β-cell mass and islet morphology

Under pathological conditions of glycemic control, alterations to β-cell mass usually occur. Another important finding of our study is the fact that alterations (especially loss) to β-cell mass can affect only *n*_β_ (a mere islet size growth or β-cell death at islet peripheral, for example), or both *n*_β_ and *n*_c_ (β-cell death across the whole islet, for example), which are independent cytoarchitectural parameters that are both important to function. Though different types of mass changes may result in the same total β-cell mass, they could nonetheless lead to distinct bursting characteristics alterations. Our simulation results indicate that a reduction in *n*_β_ that also affects *n*_c_ will modify the islet function more markedly than a reduction only in *n*_β_ (figures 5–6). To investigate the β-cell mass alterations and the consequent compromise in β-cell function, we therefore must first determine whether only *n*_β_, or both *n*_β_ and *n*_c_ are affected.

During the prediabetic development of type 2 diabetes, a transient expansion of islet size (*n*_β_↑) is often observed before β-cell mass reduction ensues [46]. Whether and how the transient change contributes to disease pathogenesis is not yet understood. During the development of type 1 diabetes, β-cell mass is gradually destroyed by infiltrating immune-cells [52], which results in a gradual reduction in both *n*_β_ and *n*_c_ for each islet. These specific characteristics in β-cell mass alteration need to be considered and differentiated when investigating each pathological condition.

### Conclusion

A quantitative understanding of the functional role of islet cytoarchitecture is much needed, but has not been investigated comprehensively till now. It is central to many important issues in the study of glycemic control and the related metabolic syndromes. Examples include the relationship between β-cell mass and β-cell function, alterations in β-cell mass under pathological conditions, the translational studies of animal islets, and the tissue engineering of islets and islet allotransplantation [53]. Specifically for a successful allotransplantation, islet preparation quality control is essential. Several commonly used parameters include the number of islets, percent of β-cell content and of non-apoptotic β cells, etc. Very recently, it has been pointed out that the morphostructural integrity of the islets, namely, the right interactions and the three-dimensional architecture among various cell populations in islets, is also critical and predictive of in vivo function and clinical outcome in islet allotransplantation [58].

Our study represents a very first step toward this goal. Much work is still needed. In an intact islet there are other islet cell types, and numerous paracrine, hormonal, and nervous signals that fine-tune the insulin secretion under different physiological conditions. The non-β cell types secrete a few other types of hormones additional to insulin. Some of the hormones, glucagon and somatostatin are clearly involved in the regulation of insulin secretion [59]. To fully understand the phenomenon of plasma insulin pulsatility, several additional levels of regulation also need to be investigated, which include but not limited to: the coupling of the β cell membrane electrical burst with glycolysis oscillation [60], and the inter-islet coordination and possible existence of a pace-making mechanism provided by the central nerves system (CNS) [61] or the intrapancreatic ganglia [62]. Our study reported here serve as a basis for these future works.

## Methods

### Mathematical model of the electrical excitability of individual β cells

Pancreatic islet β cells are electrically excitable cells. At basal level of glucose cell membranes are hyperpolarized with a resting potential around −70 mV [63], [64]. In presence of elevated plasma glucose concentration, the glucose metabolism at mitochondria results in net generation of ATP; the increased ATP:ADP ratio will close the ATP-dependent K_ATP_ channels, which will drive the membrane potential to more positive values; the membrane depolarization will trigger action potential firing and opening of the voltage-dependent Ca^2+^ channels; the influx of Ca^2+^ will raise its intracellular concentration from a basal of 0.05–0.1 µM to stimulatory values >0.15–0.2 µM that will trigger exocytosis of membrane-docked insulin granules [65], [66]. During this process, voltage–dependent K^+^ channels will open in response to membrane depolarization, and will mediate outwardly rectifying K^+^ current that serves to re-polarize cell membrane [65], [67]. Consequently, the electrical potential across β-cell membrane oscillates between depolarized plateaus, on which bursts of action potentials are superimposed, separated by repolarized electrically silent intervals. The bursting oscillations of membrane action potential give rise to oscillations in the intracellular calcium concentration and in the secretion of insulin into blood stream. The duration and the periodicity of the bursts depend on glucose concentration. The rate of insulin release is believed to be roughly proportional to the “plateau fraction”, which is defined to be the relative time spent in the active phase divided by the total burst interval [37].

Mathematically, β-cell electrical oscillation can be explained by the Hodgkin-Huxley theory of ion channel gating [68], with cell membrane electrical potential V described by a ordinary differential equation (essentially the Kirchhoff's voltage law of a RC circuit): 
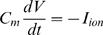
, where *C_m_* is cell membrane capacitance. Chay and Keizer were first to formulate a mathematical description of the current terms *I_ion_* for β-cells [25], based on ideas proposed by Atwater and colleagues [37]. Since then a number of modifications/extensions have been proposed [13], [15], [28], [31], [60], [69]–[71]. Here we adopt the formulation developed by Sherman et al [27], [29], for its simplicity and direct physical interpretation. For each cell *i* in a coupled β-cell cluster we have
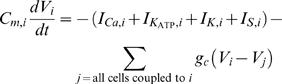
(1)where the last term is summed over all cell *j* that is coupled to cell *i*, and *g*_c_ is the inter-β cell gap junctional coupling strength. The ionic current terms include the fast voltage-dependent L-type Ca^2+^-channel current *I_Ca_*, the glucose sensitive K_ATP_ channel current 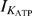, the voltage-dependent delayed rectifier K^+^ current *I_K_*, and a slow inhibitory K^+^ current *I_S_*, given by:
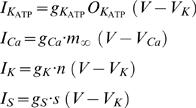
(2)where 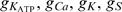 are channel conductance. The activation parameters *n*, *s* are given by
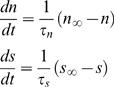
(3)with *m*_∞_, *n*_∞_, *s*_∞_ being the constants describing the fraction of open channels for Ca^2+^, fast K^+^ and slow K^+^ currents respectively at steady state, and are given by
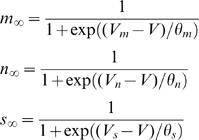
(4)The parameters *V_m_*, *V_n_*, *V_s_*, and ^*_m_*, ^*_n_*, ^*_s_* are constants that describe the dependence of channel activation on membrane voltage V. The change in intercellular calcium concentration is given by
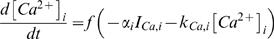
(5)where *f* is the fraction of free Ca^2+^ and *k_Ca_* is the removal rate of Ca^2+^ in the intracellular space. *α* is a conversion factor from chemical gradient to electrical gradient. Table 1 summarizes all parameters and their normal value ranges in this study.

**Table 1:** Parameters and their values.

Quantity	Description	Value (or mean value)	Unit
*C_m_*	Membrane capacitance of β-cell	5.3	pF
*r*	Radius of β-cell	6.5	µm
*g_K_*	Maximal conductance of K^+^ channel	2500	pS
*g_Ca_*	Maximal conductance of Ca^2+^ channel	1400	pS
*g_KATP_*	Maximal conductance of ATP-activated K^+^ channel	150	pS
*g_s_*	Maximal conductance of slow inhibiting K^+^ channel	200	pS
*V_K_*	Reversal Potential for K^+^ channel	−75	mV
*V_Ca_*	Reversal Potential for Ca^2+^ channel	25	mV
*α*	Conversion factor that converts electrical gradient to chemical gradient	4.5e-6	µM. fA^−1^. ms^−1^
*k_Ca_*	Rate constant for removal of intracellular Ca^2+^	0.04	ms
*f*	Fraction of free Ca^2+^ in the intracellular space	0.001	
*O_KATP_*	Fraction of free K_ATP_ channels	0.5	
*V_m_*	Half-maximal potential for the m_∞_ curve	−20	mV
*V_n_*	Half-maximal potential for the n_∞_ curve	−17	mV
*V_s_*	Half-maximal potential for the s_∞_ curve	−22	mV
*S_m_*	Slope of the m_∞_ curve at V = V_m_	12	mV
*S_n_*	Slope of the n_∞_ curve at V = V_n_	5.6	mV
*S_s_*	Slope of the h_∞_ curve at V = V_s_	8	mV
τ_sl_	Mean open time of slow inhibiting K^+^ channel	20000	ms
τ_n_	Mean open time of K^+^ channel	6.5	ms
*g_c_*	Gap junctional conductance	[25–1000]	pS

### β-cell heterogeneity

In vitro studies have suggested an extensive heterogeneity among β cells [17]. In this study we introduced independent heterogeneity for each of the parameters listed in table 2 at the level of 8–20%. The parameters of individual β cells were assumed to follow normal distribution, and were generated using the MATLAB command, ‘*normrnd*’. Membrane capacitance is proportional to the total membrane area, therefore the distribution of *C_m_* is computed from the distribution of cell radii. The heterogeneity in membrane conductance results from variations in channel density. Our approach is similar to the method outlined in [15].

**Table 2:** Heterogeneity in parameter values.

Parameters	Heterogeneity
*r*	8%
*g_Ca_*	10%
*g_K_*	10%
*g_KATP_*	20%
*g_S_*	15%
α	8%
*k_Ca_*	10%

### The HCP model of β-cell cluster

**Figure 9:**
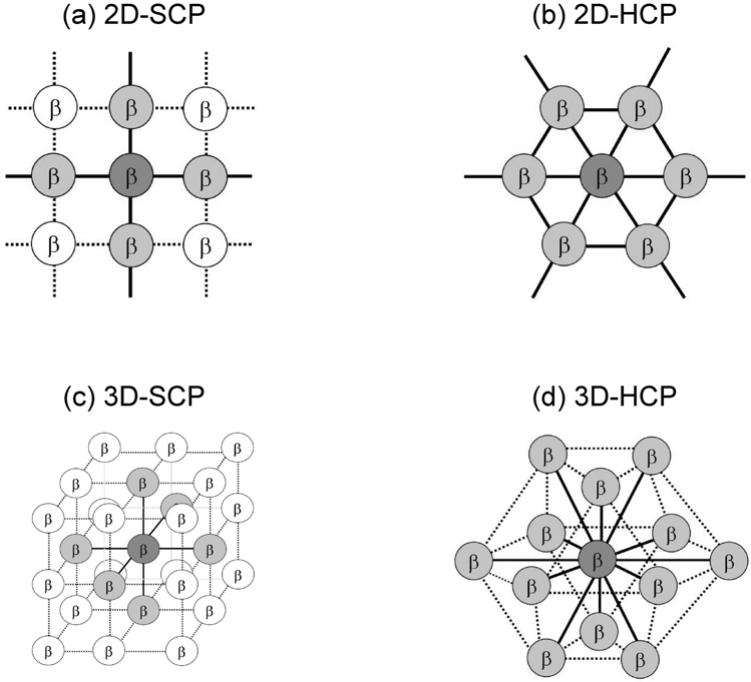
SCP and HCP β-cell clusters. In a 2D cluster *n*_c_ = 4 for SCP (a) and *n*_c_ = 6 for HCP (b). In 3D the numbers become *n*_c_ = 6 for SCP (c) and *n*_c_ = 12 for HCP (d).

In this study we introduced the HCP lattice to simulate β-cell clusters. In figure 9 we lay out the HCP structure in contrast to the SCP lattice that is conventionally used. In 2D HCP *n*_c_ = 6, a value 1.5 fold higher than in SCP. The difference is more significant in the real 3D situation, with *n*_c_ = 12 in HCP, 2 fold higher than in SCP. The number of cells in a 2D regular HCP cluster with edge size *n* is given by *N*_HCP_(*n*) = 3*n* ^2^−3*n*+1, and in a 3D HCP it is given by:
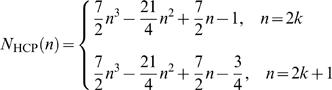
(6)


**Figure 10:**
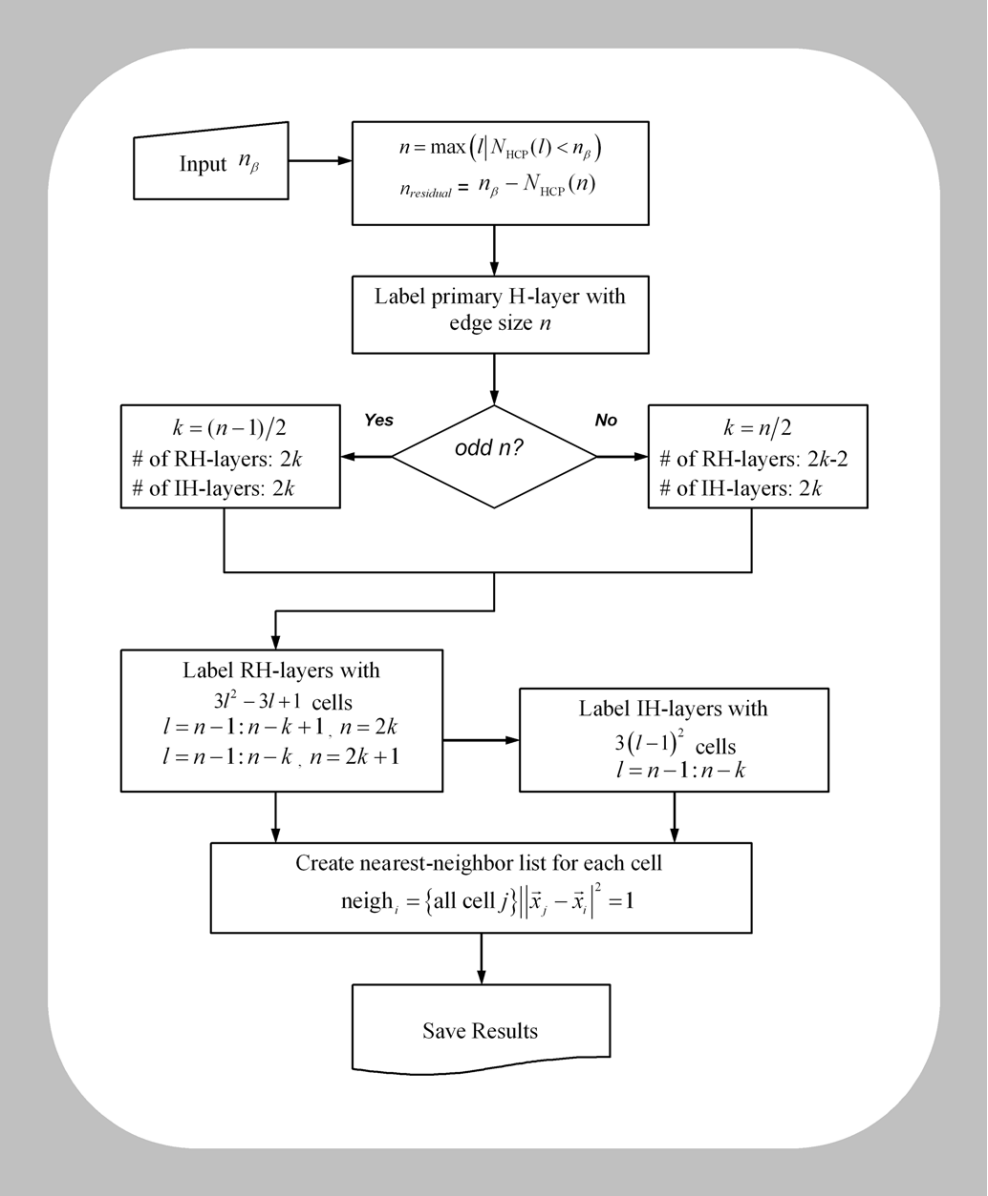
Scheme of cell labeling in an HCP β-cell cluster.

Table 3 compares the number of cells that can be packed into cubic and hexagonal lattices for a range of edge sizes *n*. To arrange β cells in an HCP cluster and setup their equations 1–5 for simulation, we have developed a cell labeling algorithm. The flowchart is presented in figure 10. Given a β-cell cluster size *n*_β_, the program will first decide according to equation 6 the maximum edge size *n* of the HCP lattice that these cells can fill up, while extra cells (*n*_β_−*N*_HCP_(*n*)) will be attached outside the complete HCP lattice in a random fashion. Labeling of the cells will start with the center layer, which we call the primary layer (figure 1). It is a 2D regular hexagon of edge size *n*, with a total of 3*n* ^2^−3*n*+1 cells. The remaining *n*−1 layers on each side (top and bottom) of the primary layer, starting from immediate layer adjacent to it, alternate between being a layer that is of irregular hexagonal shape (which we call IH layer), and being a layer that is of regular hexagonal shape (which we call RH layer). When *n* is an even number, i.e., *n* = 2*k* where *k* is an integer, the HCP cluster ends with a IH-layer on its surface; when *n* is an odd number, i.e., *n* = 2*k*+1 where *k* is an integer, the HCP ends with an RH-layer on its surface. The edge size decreases by 1 each time when traversing up or down. The number of cells in a IH layer is given by 3(n−1)^2^, and the number of cells in a RH layer is given by 3*n* ^2^−3*n*+1, where *n* is the edge size of that layer. Lastly, the program will label for each cell their nearest neighbors throughout the layers. This list will later be utilized to set up the 
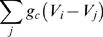
 term in equation 1 for each β cell.

**Table 3:** Number of cells that can be packed in HCP and SCP clusters

Edge length (*n*)	2D HCP	2D SCP	3D HCP	3D SCP
	*N*_HCP_(*n*) = 3*n* ^2^−3*n*+1	*N*_SCP_(*n*) = *n* ^2^	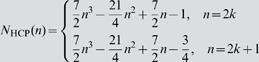	*N*_SCP_(*n*) = *n* ^3^
1	1	1	1	1
2	7	4	13	8
3	19	9	57	27
4	37	16	153	64
5	61	25	323	125
6	91	36	587	216
7	127	49	967	343
8	169	64	1483	512
9	217	81	2157	729
10	271	100	3009	1000

### Simulation details

The simulation of equations 1–5 is performed in MATLAB at time step Δ*t* = 1 ms by Euler integration, which turns out to be small enough. Simulations at smaller time steps with 0.5 and 0.2 ms were also performed for extreme conditions (the high end values of *g*_c_ = 1000 pS and *g*_c_ = 500 pS), and randomly chosen cases through out our parameter spectrum, to ensure this choice of time step. Cluster size ranged from SCP-8 and HCP-13 to SCP-343 and HCP-323 respectively. The *g*_c_ value ranged from 25 pS to 1000 pS. For each condition, 10 replicate clusters were simulated, with cells randomly generated according to parameter values listed in tables 1 and 2.

**Figure 11:**
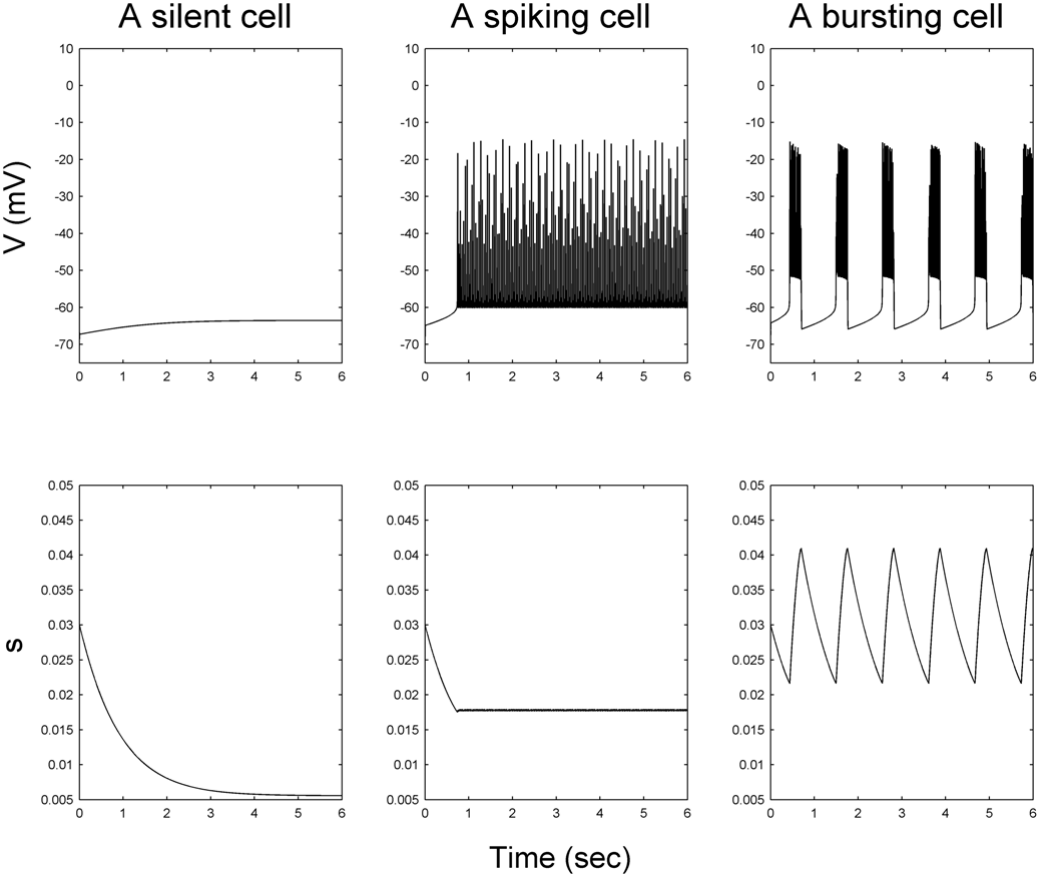
The bursting, spiking and silent cell types.

In general a β cell can be a burster cell, a spiker cell, or a silent cell. A burster cell is defined as a cell capable of producing a sequence of well-defined regular bursts which correlate with the period between the consecutive peaks and nadirs. In figure 11 we present the action potential and *s(t)* for the three types of cells respectively. Post simulation of a β-cell cluster, the oscillatory status of each β cell was first determined. Due to the scale of our simulation, this was carried out in an automatic fashion using a program that we wrote. The program first identifies the silent cells, defined to be those whose maximum and minimum membrane voltages differ less than 30 mV. For the remaining cells (which are a mixture of bursters and spikers), the peaks and nadirs of all state variables are determined by a recursive difference algorithm which extracts the peak and nadir points based on gradient change. In this study, the nadir and peak information of the *s(t)* data were used to distinguish between spikers and bursters (figure 11). Specifically, if the mean amplitude of *s* oscillation (peak-nadir) exceeds a threshold value, 15 percentile value of the maximum amplitude observed in random un-coupled β cells, the cell is considered a burster. This threshold value was determined empirically, and the algorithm has been evaluated and optimized manually using extensive simulation results.

For burster β cells, their bursting periods *T*_b_, plateau fraction *p*_f_, calcium amplitude [*Ca*], as well as degree of synchronization in bursting λ (see the next subsection) were then determined. Mean and standard deviation (SD) were also obtained for each group of 10 replicated clusters.

For clusters with *n*_β_>100 (SCP-125, -216, -343, HCP-153, -323), we have also determined the bursting properties with varying percentages (10–90%) of β cells randomly (*not from peripheral*) removed across the cluster. This way both *n*_β_ and *n*_c_ are modified, and it allows the investigation of the presence of non-β islet cells, and the loss of β-cell mass under pathological conditions. In addition to coupled β-cell clusters, 500 single β cells with the same degree of heterogeneity were simulated to provide baseline information.

### Phase synchronization analysis of bursting β cells

Laboratory measurements have established that the oscillations are nearly synchronous among the β cells within an intact islet, and this is believed to be important for its insulin release regulation [23]. In this study, we evaluate β-cell bursting synchronization within a cluster through phase synchronization analysis. It is a technique to infer interaction from the coordination and phase locking of two time series, based on analytical signal concepts [72], [73]. Compared with more traditional techniques (correlation coefficient, for example), it has the advantage of being more robust to the variation in oscillation amplitude that can mask the phase locking, and more sensitive to the inter-coordination between systems. Recently this technique was applied to the analysis of bursting synchronization between two β cells [74].

Our phase synchronization analysis of β-cell clusters was implemented in Matlab. Briefly, for each β cell *j* the instantaneous phase ϕ*_j_*(*t*) of the membrane action potential was determined through Hilbert transformation [72], [75], using the Matlab command: ϕ*_j_*(*t*) = unwrap(angle(Hilbert(detrend(*V_j_*(*t*)))). A mean field value of phase Φ is determined by taking the circular mean of the individual phase angles of all bursting β cells
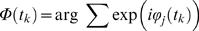
(8)The synchronization strength to mean field by each β cell can be calculated by
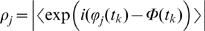
(9)A cluster synchronization index (CSI) is then defined by
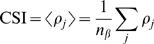
(10)When synchronization is evaluated among bursting β cells only, a simpler approach that measures the mean pair-wise phase difference can be taken. The synchronization of each pair of cells *j* and *k* is calculated by
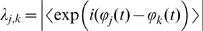
(11)The mean of all pair-wise phase difference are then determined by:
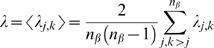
(12)

